# *Lasiodiplodia theobromae* as a causal pathogen of leaf blight, stem canker, and pod rot of *Theobroma cacao* in Malaysia

**DOI:** 10.1038/s41598-022-13057-9

**Published:** 2022-05-27

**Authors:** Abd Rahim Huda-Shakirah, Nik Mohd Izham Mohamed Nor, Latiffah Zakaria, Yin-Hui Leong, Masratul Hawa Mohd

**Affiliations:** 1 School of Biological Sciences, Universiti Sains Malaysia, 11800 Minden, Penang Malaysia; 2 National Poison Centre, Universiti Sains Malaysia, 11800 Minden, Penang Malaysia

**Keywords:** Microbiology, Molecular biology, Plant sciences

## Abstract

Symptoms of leaf blight, stem canker, and pod rot were observed on *T. cacao* during a series of samplings conducted in several states of Malaysia from September 2018 to March 2019. The identity of the pathogen that was responsible for the diseases was determined using morphological characteristics, DNA sequences, and phylogenetic analyses of multiple genes, namely, internal transcribed spacer (ITS), elongation translation factor 1-alpha (*tef1-α*), β-tubulin (*tub2*), and RNA polymerase subunit II (*rpb2*). A total of 57 isolates recovered from diseased leaves of *T. cacao* (13 isolates), stems (20 isolates), and pods (24 isolates) showed morphological features that resembled *Lasiodiplodia* sp. The identity of the isolates was further determined up to the species level by comparing DNA sequences and phylogenetic analyses of multiple genes. The phylogenetic analysis of the combined dataset of ITS, *tef1-α*, *tub2*, and *rpb2* elucidated that all of the isolates obtained were *Lasiodiplodia theobromae* as supported by 97% bootstrap value. The results of pathogenicity tests revealed *L. theobromae* as the causal pathogen of leaf blight, stem canker, and pod rot of *T. cacao*.

## Introduction

The cocoa tree (*Theobroma cacao*) is an evergreen shrub that is recognized by several names, including kakaw, pokok coklat, chocolate, cacao, koko, criollo, cacaoyer, and kakao^1^. Previously, *T. cacao* was classified under Sterculiaceae family, before being reclassified as a member of Malvaceae. It is originated in the Neotropical rainforest, particularly in the Amazon basin and on the Guyana plateau^2–4^. The word *Theobroma* means “Food of the Gods,” whereas *cacao* comes from the Mayans and Aztec languages, *Kakaw* and *Cacahuatl*, respectively^5,6^. Furthermore, *T. cacao* is the recognized species among the 22 *Theobroma* species that is commonly planted beyond its natural range and have an economic value^1,6^. Besides *T. cacoa*, the other species of *Theobroma* also have economic value such as *T. grandiflorum* in South America and *T. bicolor* in Mexico and Central America^6^. Clone seedling is preferred for plantation over hybrid seedling in almost all cocoa-producing countries because it will produce the same tree morphology, pod, and bean characteristics as the parent tree, where the clone tree has greater pod bearing capacities, bigger and more uniform beans, richer butter content, withstand to pest and pathogen attacks, and adaptable to a wide range of agro-climatic conditions^1,7^. The continued advancement of Malaysia's cocoa industry in the late 1970s and early 1980s resulted in the founding of the Malaysian Cocoa Board (MCB) in 1989, which is overseen by the Ministry of Plantation Industries and Commodities. The Board's goal was to grow Malaysia's cocoa industry so that it could be incorporated in the global market, as well as to boost the quality and performance of cocoa bean and downstream production^8^. Malaysia is now the leading country in the cocoa grinding industry^8^.

In addition, cocoa and its products have various nutritional values owing to their rich amounts of alkaloids, cardiac glycosides, catechin, enantiomer, epicatechin, flavanol, methylxanthines, procyanidin B2, saponin, tannins, and terpenoids^9^. Moreover, cocoa has several biological benefits, including high antioxidant activity, blood pressure reduction, anticancer activity, stress and depression reduction, reduced risk of heart attack and stroke, cholesterol control, antiplatelet effect, and anti-inflammatory activity^10–14^.

*Theobroma cacao* tree, similar to any other Malvaceae plants, has been shown to be fungus-prone. Among the most important diseases affecting cacao in Malaysia are black pod rot, canker, and vascular streak dieback (VSD), which affect the pod; trunk and stem; leaves and stems of the cacao tree, respectively^1^. Furthermore, several previous studies on the diseases of *T. cacao* caused by fungal and fungal-like pathogens have been reported worldwide namely, *Ceratobasidium theobromae*^15^, *Colletotrichum gloeosporioides*^6^, *Colletotrichum siamense*^16,17^, *Colletotrichum theobromicola*^18^, *Colletotrichum tropicale*^17^, *Lasiodiplodia brasiliensis*^19^, *Lasiodiplodia pseudotheobromae*^17^, *Lasiodiplodia theobromae*^6,19–25^, *Moniliophthora perniciosa*^26^*, Moniliophthora roreri*^27^, *Neofusicoccum parvum*^28^, *Phytophthora palmivora*^6,25,29^, and *Phytophthora megakarya*^4,29^.

In a series of samplings conducted from September 2018 to March 2019, the occurrences of leaf blight, stem canker, and pod rots of *T. cacao* were observed in cocoa plantations in several states of Malaysia. From observations during the sampling revealed the disease incidences of leaf blight, stem canker, and pod rots in cocoa plantations were 15%, 20%, and 25%, respectively, which may reduce cocoa production. The diseased samples were gathered and returned for further observation. Therefore, the present study sought to find the causative agent of leaf blight, stem canker, and pod rot of *T. cacao* in Malaysia using morphological, molecular, and pathogenicity analyses.

## Results

### Fungal isolation and morphological identification

**Figure 1:**
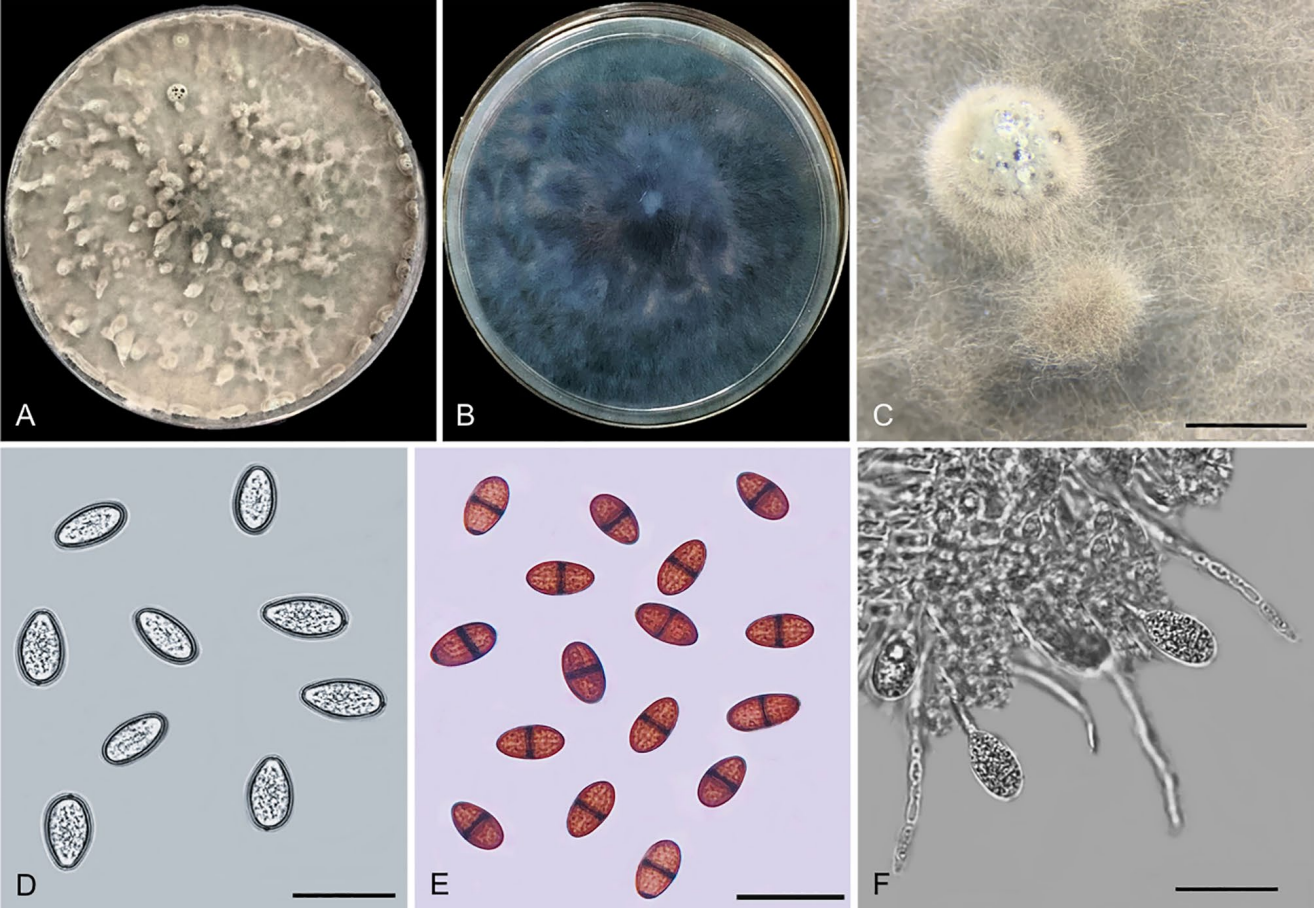
Morphological characteristics of *Lasiodiplodia* sp. recovered from diseased leaves, stem, and pods of *Theobroma cacao*. (**A**) Upper view of the colony appearance, (**B**) Reverse view colony appearance, (**C**) Conidiomata, (**D**) Immature conidia, (**E**) Mature conidia, (**F**) Conidiogenous cells and paraphyses. Scale bars: (**C**) = 1 mm; (**D**–**F**) = 50 µm.

In total, 57 fungal isolates were retrieved from diseased leaves of *T. cacao* (13 isolates), stems (20 isolates), and pods (24 isolates). On PDA, the fungal isolates produced dense and fast-growing mycelia, white to pale greenish-gray colony and eventually becoming dark grayish (Fig. 1A). The pigmentation ranged from dark gray to black (Fig. 1B). The conidiomata were solitary, globose to subglobose, uniloculate, black, surrounded by dense grayish mycelia, and 3.32 ± 0.47 × 3.10 mm ± 0.27 mm (mean ± standard deviation (SD)) (length (L) × width (W)) in size (Fig. 1C). The conidia were observed as immature and mature conidia. Both immature and mature conidia were subovoid to ellipsoid-ovoid in shape, with a broadly rounded apex and a tapering to the truncated base. The immature conidia were initially double layered, hyaline, unicellular, and 25.0 ± 1.06 × 13.0 µm ± 0.48 µm (mean ± SD) (L × W) in size (Fig. 1D). The mature conidia appeared light to dark brown color with typical striate formation, one-septate, and 25.7 ± 1.73 × 13.1 µm ± 0.82 µm (mean ± SD) (L × W) in size (Fig. 1E). The conidiogenous cells were cylindrical, hyaline, thin walled, holoblastic, and smooth. The structure of the paraphyses was aseptate and septate, with rounded apex, hyaline, and cylindrical (Fig. 1F). Based on the characterization of the morphological features of the fungal isolates, it was tentatively identified as *Lasiodiplodia* sp., which is coherent with the morphology described by Alves et al.^30^ and Phillips et al.^31^.

### Molecular identification and phylogenetic analysis

**Figure 2:**
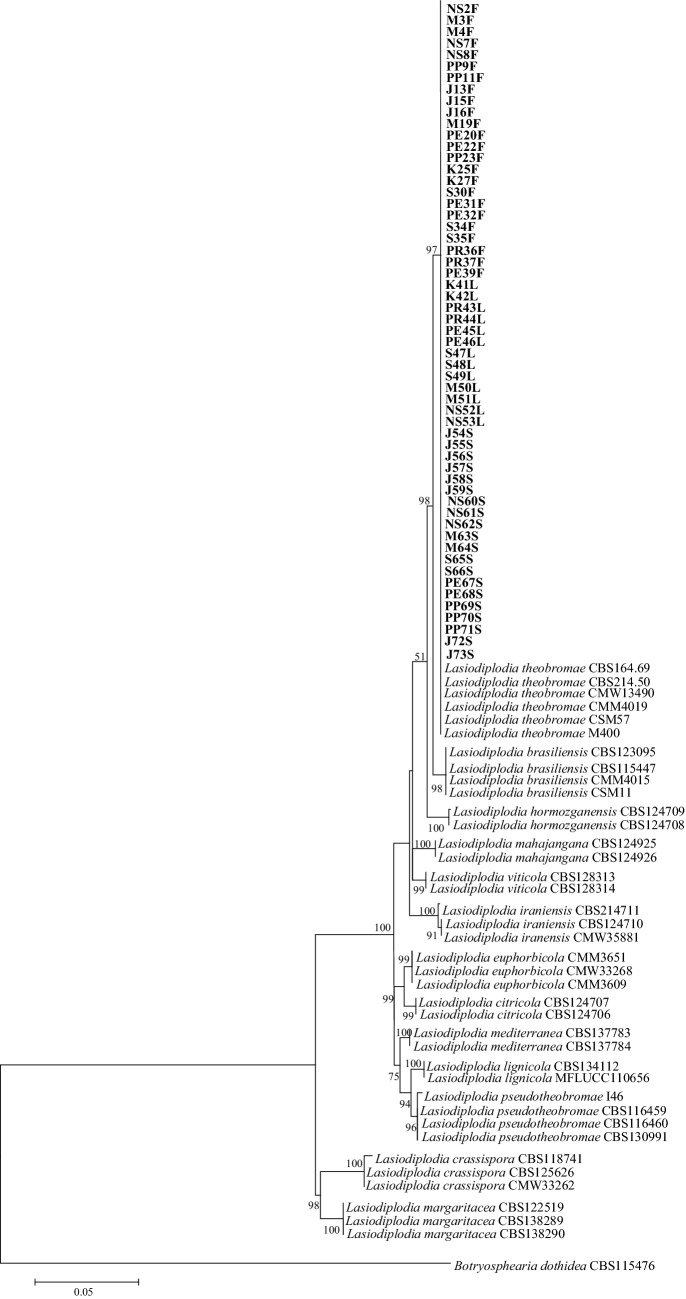
The maximum likelihood (ML) tree was generated with 1000 bootstrap replications using the Tamura-3-parameter model. The ML tree is inferred from concatenated sequence dataset of four genes (ITS, *tef1-α tub2*, and *rpb2*). Bootstrap support values greater than 50% are pointed out at the nodes. Isolates in bold represent isolates in the present study and *Botryosphearia dothidea* represents the outgroup. The bar indicates the substitutions number per position.

Molecular analysis of the sequences of ITS, *tef1-α*, *tub2*, and *rpb2* clarified the species identification of all the 57 isolates of *Lasiodiplodia* sp. recovered from *T. cacao*. BLAST searches in the GenBank database revealed that the isolates showed 98–100% sequence homology to the KY473071 (ITS), JX464026 (*tef1-α*), EU673110 (*tub2*), and MT592333 (*rpb2*) of *L. theobromae*. A multi-locus analysis was performed to explicate the phylogenetic positions of these *L. theobromae* isolates. To construct the phylogenetic tree, the sequences of the isolates from the present study (57 isolates of *L. theobromae*) were aligned with 38 reference isolates of *Lasiodiplodia* species and one outgroup taxon (*Botryosphaeria dothidea*). Phylogenetic analysis revealed that the topologies of the ML trees generated from individual and concatenated genes (ITS, *tef1-α*, *tub2*, and *rpb2*) were similar (Figs. S1a–d and 2). The ML tree constructed from the concatenated sequences confirmed that the phylogenetic positions of the 57 isolates from *T. cacao* were clustered with the reference isolates of *L. theobromae*, supported by 97% bootstrap value (Fig. 2). As a result, all the present isolates were verified as *L. theobromae* by virtue of molecular identification and phylogenetic analysis.

### Pathogenicity test

**Figure 3:**
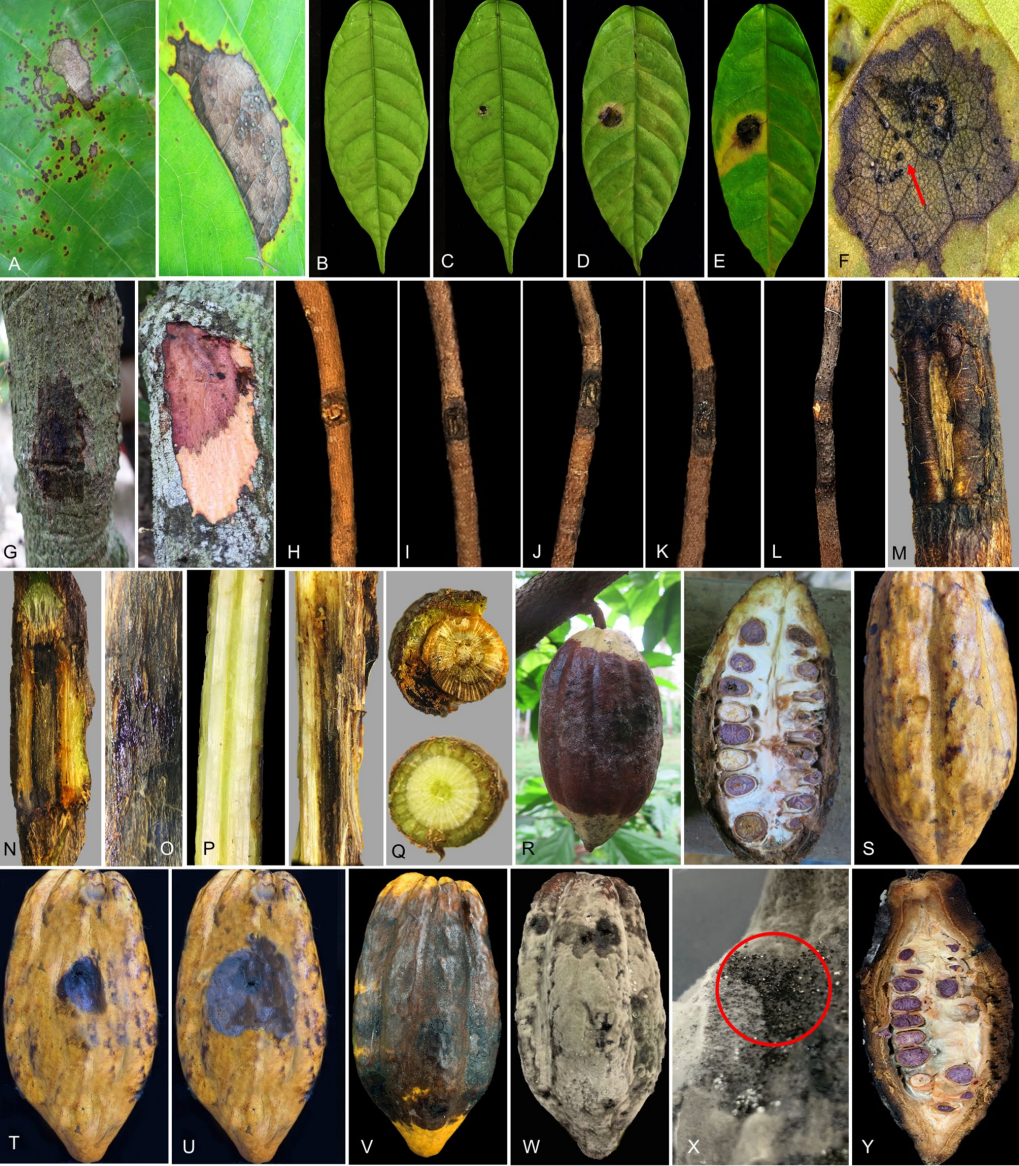
Pathogenicity of *Lasiodiplodia theobromae* on leaves, stems, and pods of *Theobroma cacao*. (**A**) Blighted leaf observed in the field, (**B**) Asymptomatic control inoculated leaf, (**C**) Irregular black lesions with yellow halo observed after 4 days of inoculation (**D**,**E**) The lesions enlarged after 6 and 9 days of inoculation, respectively, (**F**) Presence of conidiomata on the diseased area (red arrow), (**G**) Cankered stem observed in the field, (**H**) Asymptomatic control inoculated stem, (**I–K**) Black necrotic lesions observed on the inoculation sites after 7, 14, and 21 days of inoculation, respectively, (**L**) Black necrotic lesions extending upwards and downwards after 28 days of inoculation, (**M**) Black sunken lesion on the inoculation site, (**N**) Incision of the stem inoculated site showed reddish-brown to black necrotic lesion, (**O**) Formation of gummosis on the necrotic lesion, (**P**) Vertical section of control (left) and fungal inoculated stems (right) showed symptomless and dark brown to black necrotic lesion, respectively, (**Q**) Transverse section of control (below) and fungal inoculated stems (above) showed symptomless and necrotic lesion, respectively, (**R**) Rotted pod observed in the field showed external and internal rotting symptoms, (**S**) Asymptomatic control inoculated pod, (**T**) Brown to black lesions observed on the inoculation sites after 5 days of inoculation, (**U**) The lesions enlarged after 7 days of inoculation (**V**), The lesion rapidly expanded after 9 days of inoculation, (**W**) The inoculated pod completely covered by the fungal mycelia after 12 days of inoculation, (**X**) Presence of black conidiomata (red circle) on the fungal inoculated pod, (**Y**) Cross-section of fungal inoculated pod showed rotting of the internal tissue.

The pathogenicity analysis of 13, 20, and 24 fungal isolates on healthy leaves, stems, and pods of *T. cacao* resulted in the production of typical symptoms of blight, canker, and rot, respectively as observed in the fields (Fig. 3A,G,R). There were no visible symptoms produced on control points of leaves, stems, and pods (Fig. 3B,H,S).

After 4 days of inoculation, the fungal inoculated leaves exhibited small irregular black lesions bounded by yellow halos (Fig. 3C). The lesions and yellowing areas enlarged gradually during the incubation period (Fig. 3D,E). Conidiomata formed on the inoculation site (Fig. 3F). The lesion areas produced ranged from 3.0 to 4.6 cm^2^ (Table 1). There was no significant difference of lesion areas recorded among the tested isolates.

**Table 1:** Lesion area produced on the leaves, stems and pods of *Theobroma cacao* inoculated with *Lasiodiplodia theobromae*.

Isolate code	^a^Lesion area (cm^2^)		
	Leaf	Stem	Pod
K41L	3.3 ± 0.7^b^	^b^–	–
K42L	3.1 ± 0.1^b^	–	–
PR43L	3.3 ± 0.7^b^	–	–
PR44L	3.7 ± 1.0^b^	–	-
PE45L	3.3 ± 0.3^b^	–	–
PE46L	3.0 ± 0.3^b^	–	–
S47L	4.6 ± 1.2^b^	–	–
S48L	4.6 ± 1.2^b^	–	–
S49L	3.5 ± 1.0^b^	–	–
M50L	3.3 ± 0.4^b^	–	–
M51L	3.1 ± 0.3^b^	–	–
NS52L	4.0 ± 1.3^b^	–	–
NS53L	3.2 ± 0.6^b^	–	–
J54S	–	14 ± 0^d^	–
J55S	–	14 ± 0^d^	–
J56S	–	14 ± 0^d^	–
J57S	–	14 ± 0^d^	–
J58S	–	14 ± 0^d^	–
J59S	–	14 ± 0^d^	–
NS60S	–	12.3 ± 0^c^	–
NS61S	–	12.3 ± 0^c^	–
NS62S	–	14 ± 0^d^	–
M63S	–	13.1 ± 0^cd^	–
M64S	–	13.1 ± 0^cd^	–
S65S	–	13.1 ± 0^cd^	–
S66S	–	13.1 ± 0^cd^	–
PE67S	–	13.1 ± 0^cd^	–
PE68S	–	13.1 ± 0^cd^	–
PP69S	–	12.0 ± 0^c^	–
PP70S	–	12.0 ± 0^c^	–
PP71S	–	13.1 ± 0^cd^	–
J72S	–	13.1 ± 0^cd^	–
J73S	–	13.1 ± 0^cd^	–
NS2F	–	–	49.8 ± 5.3^e^
M3F	–	–	50.3 ± 3.5^e^
M4F	–	–	47.9 ± 4.0^e^
NS7F	–	–	49.2 ± 3.8^e^
NS8F	–	–	48.1 ± 4.4^e^
PP9F	–	–	48.0 ± 2.6^e^
PP11F	–	–	47.1 ± 6.8^e^
J13F	–	–	49.8 ± 7.8^e^
J15F	–	–	47.8 ± 10.1^e^
J16F	–	–	49.9 ± 7.7^e^
M19F	–	–	46.7 ± 8.0^e^
PE20F	–	–	48.3 ± 10.6^e^
PE22F	–	–	46.7 ± 8.0^e^
PP23F	–	–	49.4 ± 4.8^e^
K25F	–	–	48.3 ± 5.2^e^
K27F	–	–	47.8 ± 8.9^e^
S30F	–	–	47.2 ± 7.5^e^
PE31F	–	–	50.8 ± 12.6^e^
PE32F	–	–	49.2 ± 3.8^e^
S34F	–	–	46.8 ± 2.3^e^
S35F	–	–	46.2 ± 10.8^e^
PR36F	–	–	49.2 ± 3.8^e^
PR37F	–	–	49.6 ± 12.8^e^
PE39F	–	–	50.5 ± 7.1^e^
Control	0^a^	0^a^	0^a^

^a^Means ± standard deviation followed by different letters are significantly different (p < 0.05) according to Tukey’s test.

^b^Not applicable.

The fungal inoculated stems developed black necrotic lesions within the first to the third week of inoculation (Fig. 3I–K). After 4 weeks, the lesions extended longitudinally from the inoculation sites (Fig. 3L). The incision of the stem inoculated point displayed a reddish-brown to black necrotic lesion (Fig. 3M,N). Formation of gummosis on the necrotic lesion was also observed (Fig. 3O). Vertical and transverse sections of control and fungal inoculated stems showed symptomless and dark brown to black necrotic lesions, respectively (Fig. 3P,Q). There were significant differences of lesion areas produced on the *L. theobromae* inoculated stems that ranged from 12 to 14 cm^2^ (Table 1).

The fungal inoculated pods showed irregular brown to black lesions after 5 days of incubation (Fig. 3T). As the infection progressed, the lesions expanded and turned darker after 7 days of inoculation (Fig. 3U). After 12 days of inoculation, the lesions continued to expand, and the inoculated pods were completely colonized by the fungal grayish mycelia (Fig. 3V,W). Black conidiomata formed on the fungal inoculated pods (Fig. 3X). A cross-section of fungal inoculated pods showed rotting of the internal tissue (Fig. 3Y). The lesion areas ranged from 46.7 to 50.3 cm^2^ (Table 1). The lesion areas recorded on the fungal inoculated pods were significantly different compared to the control (Table 1).

The repetition of the pathogenicity assessment yielded the same outcomes as the first analysis. Koch's postulates were achieved by reisolating the same fungal isolates from the symptomatic inoculated leaves, stems, and pods of *T. cacao* and their identities were confirmed through morphological features.

## Discussion

The present study identified *L. theobromae* isolates responsible to cause leaf blight, stem canker, and pod rot of *T. cacao* in Malaysia based on the morphological features, sequence comparison, and phylogenetic analysis of four genes (ITS, *tef1-α*, *tub2*, and *rpb2*). Fungi from genus *Lasiodiplodia* are cosmopolitan and belong to the Botryosphaeriaceae family, and most of the species can be primarily found in tropics and subtropics^31–33^. The genus consists of many phytopathogenic fungal species with widespread distribution^33^. *Lasiodiplodia* species responsible to cause over 500 plant diseases, including fruit rot, root rot, collar rot, stem-end rot, dieback, canker, and leaf necrosis^32,34–43^. In Malaysia, *Lasiodiplodia* species have been attributed to various destructive diseases, such as black rot of kenaf seeds^44^, leaf blight of *Sansevieria trifasciata*^45^, stem end-rot of *Mangifera indica*^46^, stem canker on *Jatropha curcas* and *Acacia mangium*^47,48^, and fruit rot of mango and guava^49,50^. Apart from that, they can act as secondary pathogens or endophytes, and they also can become pathogenic in response to a stressor^34,36,40^.

All the 57 fungal isolates recovered from diseased *T. cacao* in the present study was tentatively assigned as *Lasiodiplodia* sp. based on their macroscopic and microscopic characteristics. According to Hyde et al.^51^, the morphological approach has been widely used as the foundation for almost all studies of fungal taxonomy. Slippers and Wingfield^34^ also stated that Botryosphaeriaceae members are easily recognized from most other fungi through their colony appearance, aerial mycelium, and pigments, which can aid in the delimitation and rapid identification. However, due to the significant overlapping of key morphological characteristics among *Lasiodiplodia* species, clear-cut identification of the *Lasiodiplodia* isolates in the present study could not be achieved up to the species level by using traditional morphological descriptions such as conidial shape^30,40^.

Attributable to unresolve identity of *Lasiodiplodia* isolates based on morphological characteristics that could lead to uncertain and misleading results, phylogenetic analysis involving DNA sequences of multiple genes was applied to delineate species boundaries. Consistent with previous studies that also highlighted the importance of molecular work in defining *Lasiodiplodia* species^34,39,40,52^, the present study used several genes, namely, ITS, *tef1-α*, *tub2*, and *rpb2*, to explicitly characterize *Lasiodiplodia* isolates. The ITS region has been proposed and widely used in fungal taxonomic classification because of its straightforward amplification and it provides a high probability of successful fungal recognition, with the barcoding difference between inter- and intraspecific variations^53,54^. Nonetheless, the ITS region lacks interspecies variety and may even be vague in the identification of some fungi, thus the use of additional genes would provide better resolution in the phylogenetic analysis. Other studies also showed that a single gene is incapable of determining species in the genus *Lasiodiplodia*, implying that additional genes are required^30,55^. The *tef1-α* has become the marker of choice for fungal identification because of its distinct polymorphisms among similar species and consists of non-orthologous copies of the gene that are undetected in the genus^56^. The *tub2* is another useful marker for delineating fungal species because it has fewer obscure aligned regions and less homoplasy across genera^57^. The *rpb2* gene which codes for the second-largest protein subunit in fungi is a highly preserved single-copy gene^54^.

According to the results of phylogenetic analysis, it can be inferred that single gene analyses of ITS, *tub2*, and *rpb2* are unable to resolve the identity of *Lasiodiplodia* isolates in the present study (Fig. S1a,c,d)*.* Those phylogenetic trees displayed that *L. theobromae* was grouped with *L. brasiliensis* and *L. hormozganensis*. On the contrary, phylogenetic analysis of *tef1-α* sequences was able to differentiate isolates in the present study with other species of *Lasiodiplodia* by clustering them with several reference sequences of *L. theobromae* from the GenBank database with only 64% bootstrap value (Fig. S1b). Owing to the fact that single gene analysis could not accurately identify the *Lasiodiplodia* isolates in the present study, the combination of ITS, *tef1-α*, *tub2*, and *rpb2* sequences was used for better characterization. The phylogenetic inferences based on multiple gene sequences revealed that the present isolates were grouped with *L. theobromae* with a higher bootstrap value (97%) (Fig. 2). The finding has been proven that phylogenetic analysis based on multigene provided robust resolution with clear-cut fungal identity. This is in line with the findings of Cruywagen et al.^52^.

*Lasiodiplodia theobromae* was confirmed to be the causal pathogen of leaf blight, stem canker, and pod rot of *T. cacao* in Malaysia. In 1895, *L. theobromae* was firstly described and reported to cause minor charcoal rot on cocoa in Ecuador^31^. Besides charcoal rot, *L. theobromae* was also reported to cause dieback on *T. cacao* since the late 1980s^20^. In Malaysia, documentations of relationship between *L. theobromae* and *T. cacao* are still limited. The present study represents the first report of leaf blight, stem canker, and pod rot of *T. cacao* caused by *L. theobromae*. Several studies have also found the incidence of *L. theobromae* causing foliar diseases in a wide range of hosts, including *Camellia sinensis*^42^, *Catasetum fimbriatum*^58^, *Cocos nucifera*^59,60^, *Kadsura longipedunculata*^61^, and *S. trifasciata*^45^. Moreover, the present study also revealed the ability of *L. theobromae* isolates to cause stem canker of *T. cacao*. Asman et al.^24^, previously reported *L. theobromae* as a causal agent of dieback and stem canker of cocoa by demonstrating internal discoloration with visible brown streaks in the vascular cambium. Furthermore, *L. theobromae* has been associated with cocoa dieback in Cameroon, India, and Venezuela^19–21^. It also responsible to cause dieback and stem canker on a number of plants, such as American ash (*Fraxinus americana*)^62^, blueberry bushes (*Vaccinium* spp.)^63^, strawberry (*Fragaria* × *ananassa*)^41^, mango (*M. indica*)^64^, cashew (*Anacardium occidentale*)^65^, sacha inchi (*Plukenetia volubilis*)^66^, Persian lime (*Citrus latifolia*)^67^, and grapevine (*Vitis vinifera*)^68^. In addition to infecting the leaf and stem, cocoa pod was also found to be susceptible to *L. theobromae* infection by showing rot symptoms. Several studies reported the occurrence of pod rot of *T. cacao* caused by *L. theobromae*^6,22,25^. Other pathogens were also identified to cause the same disease on the cocoa pod, namely *C. gloeosporioides*^6^, *C. siamense*^17^, *C. tropicale*^17^, *L. pseudotheobromae*^17^, *N. parvum*^25^, *P. palmivora*^6,25,29^, and *P. megakarya*^4,29^. From the pathogenicity tests, isolates of *L. theobromae* required wound to initiate infection and colonization on the host plant. Other studies have found that fungi from Botryosphaeriaceae can invade plants via endophytic conquest, injuries, seed-to-seedling conquest, contaminated soil, and insect infestation^34,36^.

In conclusion, the current study emphasized the first report of *L. theobromae* as a causal pathogen of leaf blight, stem canker, and pod rot of *T. cacao* in Malaysia. The pathogen was identified using morphological features supported by multigene DNA sequences and phylogenetic inference. The valid and precise identification of phytopathogen is critical for quarantine purpose and disease management strategies.

## Materials and methods

### Collecting samples and isolating fungi

**Figure 4:**
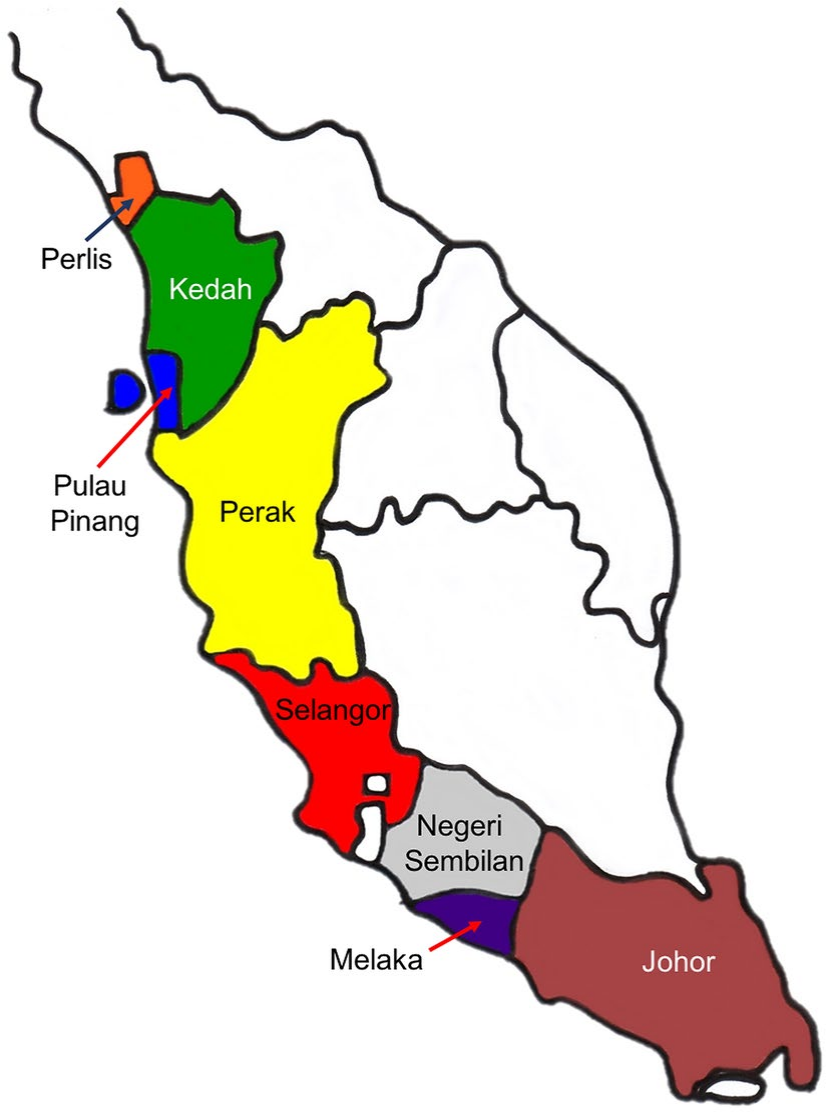
Sampling sites of diseased *Theobroma cacao* in several states of Malaysia.

From September 2018 to March 2019, sampling was conducted during rainy season in several states of Malaysia, including Johor, Kedah, Melaka, Negeri Sembilan, Perak, Perlis, Pulau Pinang, and Selangor (Fig. 4). The sampling sites and sampling activities were approved by the MCB comply with relevant institutional, national, and international guidelines and legislation. During the sampling, 50 blighted leaves, cankered stems, and rotted pods of *T. cacao* from the Koko Mardi (KM) clone were collected. The clone was used in the study because of its wide cultivation in Malaysia which showed susceptibility to a number of fungal diseases. Symptomatic leaves showed blighted symptoms, including circular to irregular blackish lesion surrounded by a yellow halo. The cankered stems were characterized as irregular blackish lesion, sometimes accompanied by gummosis on the disease area, expanded longitudinally, and internally became reddish-brown. The rotted pods were associated with dark brown to blackish lesions on the pods that eventually expanded and rotted.

The diseased and healthy margins of samples were cut into small pieces for fungal isolation. The small pieces of samples were surface-sterilized in 70% ethanol (C_2_H_5_OH) and 1% sodium hypochlorite (NaOCl) separately for 3 min. The samples were then rinsed in sterile distilled water three times in succession for 1 min each. The sterilized sample was blotted dry on sterile filter paper, transferred onto potato dextrose agar (PDA), and incubated at 25 °C ± 2 °C for 3–5 days. Pure cultures of fungal isolates obtained from single spore isolation were used for morphological and molecular assessments.

### Morphological identification

In the present study, the fungal isolates obtained were provisionally examined based on morphological features, specifically macroscopic and microscopic characteristics. Colony appearance and pigmentation were observed at the macroscopic level. Under a dissecting microscope, the structure of the conidiomata was observed and photographed (EZ4, Leica Microsystem, Germany). The microscopic features such as conidia, conidiogenous cells, and paraphyses were observed using a light microscope (CX41, Olympus, Japan) and a camera (KY-F55BE, JVC, Japan). The average size of 30 randomized conidia was measured and recorded. Each fungal isolate was cultured onto carnation leaf agar (CLA) and incubated at 25 °C ± 2 °C for 7 days to observe the structures of conidiomata, conidia, conidiogenous cells, and paraphyses.

### Molecular identification and phylogenetic analysis

To corroborate the identity of the fungal isolates of the present study, molecular identification and characterization was carried out. The fungal isolates were cultured in potato dextrose broth (PDB) and subjected to incubation at 25 °C ± 2 °C for 5 to 7 days. The mycelia that grew on the surface of PDB were collected, placed on the sterile filter paper (Whatman No. 1), and left to dry for 10 min. Using a sterile mortar and pestle, the dried mycelia were ground to a fine powder in liquid nitrogen. Then, 0.05 g of the fine powdered mycelia was placed in a 1.5 ml microcentrifuge tube for DNA extraction. The InnuPREP Plant DNA kit (Analytik Jena, Germany) was used to extract DNA by referring to the manufacturer's protocols. For amplification of internal transcribed spacer (ITS), elongation translation factor 1-alpha (*tef1-α*), β-tubulin (*tub2*), and RNA polymerase subunit II (*rpb2*), primer pairs of ITS1 (TCCGTAGGTGAACCTGCGG)/ITS4 (TCCTCCGCTTATTGATATGC)^69^, EF1-688F (CGGTCACTTGATCTACAAGTGC)/EF1-1251R (CCTCGAACTCACCAGTACCG)^30^, Bt2a (GGTAACCAAATCGGTGCTGCTTTC)/Bt2b (ACCCTCAGTGTAGTGACCCTTGGC)^70^, and *rpb2*-LasF (GGTAGCGACGTCACTCCT)/*rpb2*-LasR (GCGCAAATACCCAGAATCAT)^52^ were adopted, respectively. A reaction mixture of 50 µl was prepared by adding 8 µl of green buffer (Promega, USA), 8 µl of MgCl_2_ (Promega, USA), 1 µl of deoxynucleotide triphosphate polymerase (dNTP) (Promega, USA), 8 µl of each primer (Promega, USA), 0.3 µl of Taq polymerase (Promega, USA), 1 µl of genomic DNA, and sterile distilled water to obtain a total volume of 50 µl. The following conditions were used in the polymerase chain reaction (PCR) with the MyCycler™ Thermal Cycler (Bio-rad, Hercules, USA): Initial denaturation at 95 °C for 7 min (ITS)/5 min (*tef1-α* and *tub2*)/2 min (*rpb2*), then 25 cycles (ITS)/30 cycles (*tef1-α* and *tub2*)/35 cycles (*rpb2*) of denaturation at 94 °C for 1 min (ITS)/30 s (*tef1-α*, *tub2*, and *rpb2*), annealing at 50 °C for 1 min (ITS)/55 °C for 45 s (*tef1-α* and *tub2*)/54 °C for 30 s (*rpb2*), extension at 72 °C for 1 min (ITS and *rpb2*)/90 s (*tef1-α* and *tub2*), and final extension at 72 °C for 10 min (ITS, *tef1-α*, and *tub2*)/8 min (*rpb2*). The PCR products were electrophoresed for 90 min at 80 V and 400 mA in a 1.0% agarose gel (Promega, USA) containing FloroSafe DNA stain (First Base) in a 1.0× Tris–borate EDTA buffer. The Bio-Rad Molecular Imager^®^ Gel Doc™ XR System and Bio-Rad Quantity One^®^ Software were used to view and photograph the gel. The size of the amplified PCR products was determined using a 100 bp GeneRulers™ DNA ladder (Thermo Scientific, USA). The PCR products were sent to the First BASE Laboratories Sdn Bhd in Seri Kembangan, Malaysia, for DNA purification and sequencing.

The sequences obtained were compared, and phylogenetic analysis was performed using the Molecular Evolutionary Genetic Analysis (MEGA7) software^71^. The nucleotide homogeneity of the resulting consensus sequences was assessed by comparing with other sequence data in the GenBank database using Basic Local Alignment Search Tools (BLAST) (https://blast.ncbi.nlm.nih.gov/Blast.cgi). All sequences obtained were submitted to the GenBank database. Table 2 lists the sequences from the present study and the reference isolates used for phylogenetic analysis. The phylogenetic classification of the isolates from the present study was performed by analyzing the combination of multi-sequence alignments of fungal isolates and reference isolates in MEGA7 using the maximum likelihood (ML) method. The ML tree of combined genes was constructed using the Tamura 3-parameter model^72^ and 1000 bootstrap replicates^73^.

**Table 2:** List of GenBank accession numbers of *Lasiodiplodia* species and the outgroup (*Botryosphearia dothidea)* used in the phylogenetic analysis.

Species	Isolate	Host	Location	GenBank accession number				References
				ITS	*tef1-α*	*tub2*	*rpb2*	
*Lasiodiplodia brasiliensis*	CBS123095	*Theobroma cacao*	Cameroon	MT587423	MT592135	MT592615	MT592309	Zhang et al.^75^
*L. brasiliensis*	CBS115447	*Psychotria tutcheri*	Hong Kong	MT587422	MT592134	MT592614	MT592308	Zhang et al. ^75^
*L. brasiliensis*	CMM4015^a^	*Mangifera indica*	Brazil	JX464063	JX464049	MT592614	MT592308	Marques et al.^76^
*L. brasiliensis*	CSM11	*Theobroma cacao*	Venezuela	MF436018	MF436006	MF435998	MT592308	Mohali-Castillo and Stewart^19^
*Lasiodiplodia citricola*	CBS124707^a^	*Citrus* sp.	Iran	GU945354	GU945340	KU887505	KU696351	Cruywagen et al.^52^; Abdollahzadeh et al.^55^
*L. citricola*	CBS124706	*Citrus* sp.	Iran	GU945353	GU945339	KU887504	KU696350	Cruywagen et al.^52^; Abdollahzadeh et al.^55^
*Lasiodiplodia crassispora*	CBS118741^a^	*Santalum album*	Australia	DQ103550	DQ103557	KU887506	KU696353	Cruywagen et al.^52^
*L. crassispora*	CBS125626	*Vitis vinifera*	South Africa	MT587424	DQ103557	MT592617	MT592312	Zhang et al.^75^
*L. crassispora*	CMW33262	*Adansonia* sp.	Unknown	KU887068	DQ103557	KU887426	KU887364	Cruywagen et al.^52^
*Lasiodiplodia euphorbiicola*	CMM3609^a^	*Jatropha curcas*	Brazil	KF234543	KF226689	KF254926	KU887367	Machado et al.^77^
*L. euphorbiicola*	CMM3651	*Jatropha curcas*	Brazil	KF234553	KF226711	KF254937	KU887367	Machado et al.^77^
*L. euphorbiicola*	CMW33268	*Adansonia* sp.	Unknown	KU887131	KU887008	KU887430	KU887367	Cruywagen et al.^52^
*Lasiodiplodia hormozganensis*	CBS124709^a^	*Olea* sp.	Iran	GU945355	GU945343	KU887515	KU696361	Cruywagen et al.^52^; Abdollahzadeh et al.^55^
*L. hormozganensis*	CBS124708	*Mangifera indica*	Iran	GU945356	GU945344	KU887514	KU696360	Cruywagen et al.^52^; Abdollahzadeh et al.^55^
*Lasiodiplodia iraniensis*	CBS124710^a^	*Salvadora persica*	Iran	GU945348	GU945336	KU887516	KU696363	Cruywagen et al.^52^; Abdollahzadeh et al.^55^
*L. iraniensis*	CBS124711	*Juglans* sp.	Iran	GU945347	GU945335	KU887517	KU696362	Cruywagen et al.^52^; Abdollahzadeh et al.^55^
*L. iraniensis*	CMW35881	*Adansonia* sp.	Unknown	KU887092	KU886970	KU887464	KU887388	Cruywagen et al.^52^
*Lasiodiplodia lignicola*	CBS134112^a^	Dead wood	Thailand	JX646797	KU887003	JX646845	KU696364	Cruywagen et al.^52^; Liu et al.^78^
*L. lignicola*	MFLUCC110656	Dead wood	Thailand	JX646798	KU887003	JX646846	KU696364	Cruywagen et al.^52^; Liu et al.^78^
*Lasiodiplodia mahajangana*	CBS124925^a^	*Terminalia catappa*	Madagascar	FJ900595	FJ900641	KU887518	KU696365	Cruywagen et al.^52^; Begoude et al.^79^
*L. mahajangana*	CBS124926	*Terminalia catappa*	Madagascar	FJ900596	FJ900642	KU887519	KU696366	Cruywagen et al.^52^; Begoude et al.^79^
*Lasiodiplodia margaritacea*	CBS122519^a^	*Adansonia gibbosa*	Australia	EU144050	EU144065	KU887520	KU696367	Cruywagen et al.^52^
*L. margaritacea*	CBS138289	*Combretum elaeagnoides*	Namibia	KP872320	KP872349	KP872379	KP872429	Zhang et al.^75^
*L. margaritacea*	CBS138290	*Combretum collinum*	Zambia	KP872321	KP872350	KP872380	KP872430	Zhang et al.^75^
*Lasiodiplodia mediterranea*	CBS137783^a^	*Quercus ilex*	Italy	KJ638312	KJ638331	KU887521	KU696368	Cruywagen et al.^52^; Linaldeddu et al.^80^
*L. mediterranea*	CBS137784	*Vitis vinifera*	Italy	KJ638311	KJ638330	KU887522	KU696369	Cruywagen et al.^52^; Linaldeddu et al.^80^
*Lasiodiplodia pseudotheobromae*	CBS116459^a^	*Gmelina arborea*	Costa Rica	EF622077	EF622057	EU673111	KU696376	Alves et al.^30^; Phillips et al.^81^
*L. pseudotheobromae*	CBS116460	*Acacia mangium*	Costa Rica	MT587433	MT592145	KU198428	MT592322	Zhang et al.^75^
*L. pseudotheobromae*	CBS130991	*Mangifera indica*	Egypt	MT587433	MT592145	MT592629	MT592325	Zhang et al.^75^
*L. pseudotheobromae*	I46	*Theobroma cacao*	Puerto Rico	MK693211	MK693707	MK693702	KU696376	Serrato-Diaz et al.^17^
*Lasiodiplodia theobromae*	CBS164.69^a^	Fruit on coral reef coast	Indonesia: New Guinea	AY640255	AY640258	EU673110	KU696383	Cruywagen et al.^52^
*L. theobromae*	CBS214.50	*Cajanus cajan*	India	MT587440	MT592152	MT592637	MT592333	Zhang et al.^75^
*L. theobromae*	CMW13490	*Eucalyptus urophylla*	Venezuela: Acarigua	KY473071	KY473019	KY472962	KY472888	Mehl et al.^82^
*L. theobromae*	CMM4019	*Mangifera indica*	Brazil	JX464096	JX464026	EU673110	KU696383	Marques et al.^76^
*L. theobromae*	CSM57	*Theobroma cacao*	Venezuela	MF436029	MF436017	MF435999	KU696383	Mohali-Castillo and Stewart^19^
*L. theobromae*	M400	*Theobroma cacao*	USA: Puerto Rico	MN446021	MN536705	MN536694	KU696383	Puig et al.^25^
*L. theobromae*	NS2F	*Theobroma cacao*	Malaysia: Negeri Sembilan	OL831055	OL863319	OL863262	OL863376	This study
*L. theobromae*	M3F	*Theobroma cacao*	Malaysia: Melaka	OL831056	OL863320	OL863263	OL863377	This study
*L. theobromae*	M4F	*Theobroma cacao*	Malaysia: Melaka	OL831057	OL863321	OL863264	OL863378	This study
*L. theobromae*	NS7F	*Theobroma cacao*	Malaysia: Negeri Sembilan	OL831058	OL863322	OL863265	OL863379	This study
*L. theobromae*	NS8F	*Theobroma cacao*	Malaysia: Negeri Sembilan	OL831059	OL863323	OL863266	OL863380	This study
*L. theobromae*	PP9F	*Theobroma cacao*	Malaysia: Pulau Pinang	OL831060	OL863324	OL863267	OL863381	This study
*L. theobromae*	PP11F	*Theobroma cacao*	Malaysia: Pulau Pinang	OL831061	OL863325	OL863268	OL863382	This study
*L. theobromae*	J13F	*Theobroma cacao*	Malaysia: Johor	OL831062	OL863326	OL863269	OL863383	This study
*L. theobromae*	J15F	*Theobroma cacao*	Malaysia: Johor	OL831063	OL863327	OL863270	OL863384	This study
*L. theobromae*	J16F	*Theobroma cacao*	Malaysia: Johor	OL831064	OL863328	OL863271	OL863385	This study
*L. theobromae*	M19F	*Theobroma cacao*	Malaysia: Melaka	OL831065	OL863329	OL863272	OL863386	This study
*L. theobromae*	PE20F	*Theobroma cacao*	Malaysia: Perak	OL831066	OL863330	OL863273	OL863387	This study
*L. theobromae*	PE22F	*Theobroma cacao*	Malaysia: Perak	OL831067	OL863331	OL863274	OL863388	This study
*L. theobromae*	PP23F	*Theobroma cacao*	Malaysia: Pulau Pinang	OL831068	OL863332	OL863275	OL863389	This study
*L. theobromae*	K25F	*Theobroma cacao*	Malaysia: Kedah	OL831069	OL863333	OL863276	OL863390	This study
*L. theobromae*	K27F	*Theobroma cacao*	Malaysia: Kedah	OL831070	OL863334	OL863277	OL863391	This study
*L. theobromae*	S30F	*Theobroma cacao*	Malaysia: Selangor	OL831071	OL863335	OL863278	OL863392	This study
*L. theobromae*	PE31F	*Theobroma cacao*	Malaysia: Perak	OL831072	OL863336	OL863279	OL863393	This study
*L. theobromae*	PE32F	*Theobroma cacao*	Malaysia: Perak	OL831073	OL863337	OL863280	OL863394	This study
*L. theobromae*	S34F	*Theobroma cacao*	Malaysia: Selangor	OL831074	OL863338	OL863281	OL863395	This study
*L. theobromae*	S35F	*Theobroma cacao*	Malaysia: Selangor	OL831075	OL863339	OL863282	OL863396	This study
*L. theobromae*	PR36F	*Theobroma cacao*	Malaysia: Perlis	OL831076	OL863340	OL863283	OL863397	This study
*L. theobromae*	PR37F	*Theobroma cacao*	Malaysia: Perlis	OL831077	OL863341	OL863284	OL863398	This study
*L. theobromae*	PE39F	*Theobroma cacao*	Malaysia: Perak	OL831078	OL863342	OL863285	OL863399	This study
*L. theobromae*	K41L	*Theobroma cacao*	Malaysia: Kedah	OL831081	OL863343	OL863286	OL863400	This study
*L. theobromae*	K42L	*Theobroma cacao*	Malaysia: Kedah	OL831082	OL863344	OL863287	OL863401	This study
*L. theobromae*	PR43L	*Theobroma cacao*	Malaysia: Perlis	OL831083	OL863345	OL863288	OL863402	This study
*L. theobromae*	PR44L	*Theobroma cacao*	Malaysia: Perlis	OL831084	OL863346	OL863289	OL863403	This study
*L. theobromae*	PE45L	*Theobroma cacao*	Malaysia: Perak	OL831085	OL863347	OL863290	OL863404	This study
*L. theobromae*	PE46L	*Theobroma cacao*	Malaysia: Perak	OL831086	OL863348	OL863291	OL863405	This study
*L. theobromae*	S47L	*Theobroma cacao*	Malaysia: Selangor	OL831087	OL863349	OL863292	OL863406	This study
*L. theobromae*	S48L	*Theobroma cacao*	Malaysia: Selangor	OL831088	OL863350	OL863293	OL863407	This study
*L. theobromae*	S49L	*Theobroma cacao*	Malaysia: Selangor	OL831089	OL863351	OL863294	OL863408	This study
*L. theobromae*	M50L	*Theobroma cacao*	Malaysia: Melaka	OL831090	OL863352	OL863295	OL863409	This study
*L. theobromae*	M51L	*Theobroma cacao*	Malaysia: Melaka	OL831091	OL863353	OL863296	OL863410	This study
*L. theobromae*	NS52L	*Theobroma cacao*	Malaysia: Negeri Sembilan	OL831080	OL863354	OL863297	OL863411	This study
*L. theobromae*	NS53L	*Theobroma cacao*	Malaysia: Negeri Sembilan	OL831079	OL863355	OL863298	OL863412	This study
*L. theobromae*	J54S	*Theobroma cacao*	Malaysia: Johor	OL831092	OL863356	OL863299	OL863413	This study
*L. theobromae*	J55S	*Theobroma cacao*	Malaysia: Johor	OL831093	OL863357	OL863300	OL863414	This study
*L. theobromae*	J56S	*Theobroma cacao*	Malaysia: Johor	OL831094	OL863358	OL863301	OL863415	This study
*L. theobromae*	J57S	*Theobroma cacao*	Malaysia: Johor	OL831095	OL863359	OL863302	OL863416	This study
*L. theobromae*	J58S	*Theobroma cacao*	Malaysia: Johor	OL831096	OL863360	OL863303	OL863417	This study
*L. theobromae*	J59S	*Theobroma cacao*	Malaysia: Johor	OL831097	OL863361	OL863304	OL863418	This study
*L. theobromae*	NS60S	*Theobroma cacao*	Malaysia: Negeri Sembilan	OL831098	OL863362	OL863305	OL863419	This study
*L. theobromae*	NS61S	*Theobroma cacao*	Malaysia: Negeri Sembilan	OL831099	OL863363	OL863306	OL863420	This study
*L. theobromae*	NS62S	*Theobroma cacao*	Malaysia: Negeri Sembilan	OL831100	OL863364	OL863307	OL863421	This study
*L. theobromae*	M63S	*Theobroma cacao*	Malaysia: Melaka	OL831101	OL863365	OL863308	OL863422	This study
*L. theobromae*	M64S	*Theobroma cacao*	Malaysia: Melaka	OL831102	OL863366	OL863309	OL863423	This study
*L. theobromae*	S65S	*Theobroma cacao*	Malaysia: Selangor	OL831103	OL863367	OL863310	OL863424	This study
*L. theobromae*	S66S	*Theobroma cacao*	Malaysia: Selangor	OL831104	OL863368	OL863311	OL863425	This study
*L. theobromae*	PE67S	*Theobroma cacao*	Malaysia: Perak	OL831105	OL863369	OL863312	OL863426	This study
*L. theobromae*	PE68S	*Theobroma cacao*	Malaysia: Perak	OL831106	OL863370	OL863313	OL863427	This study
*L. theobromae*	PP69S	*Theobroma cacao*	Malaysia: Pulau Pinang	OL831107	OL863371	OL863314	OL863428	This study
*L. theobromae*	PP70S	*Theobroma cacao*	Malaysia: Pulau Pinang	OL831108	OL863372	OL863315	OL863429	This study
*L. theobromae*	PP71S	*Theobroma cacao*	Malaysia: Pulau Pinang	OL831109	OL863373	OL863316	OL863430	This study
*L. theobromae*	J72S	*Theobroma cacao*	Malaysia: Johor	OL831110	OL863374	OL863317	OL863431	This study
*L. theobromae*	J73S	*Theobroma cacao*	Malaysia: Johor	OL831111	OL863375	OL863318	OL863432	This study
*Lasiodiplodia viticola*	CBS128313^a^	hybrid grape Vignoles	USA	HQ288227	HQ288269	HQ288306	KU696385	Cruywagen et al.^52^
*L. viticola*	CBS128314	Chardonel	USA	HQ288228	HQ288270	HQ288307	KU696386	Cruywagen et al.^52^
*Botryosphearia dothidea*	CBS115476	*Prunus* sp.	Switzerland	KF766151	AY236898	MT592470	DQ677944	Slippers et al.^83^

^a^Ex-type isolates.

### Pathogenicity tests

A total of 57 fungal isolates were assessed for pathogenicity on leaves (13 isolates), stems (20 isolates), and pods (24 isolates) of *T. cacao* using KM clone. The 1-year-old healthy seedlings of *T. cacao* grown using clay loam soil with a pH of 6.5–7 in polythene bags; and healthy mature pods (5 months old and 17 cm in size) taken from 3-year-old trees were purchased from the MCB. The seedlings were placed in the plant house of the School of Biological Sciences, Universiti Sains Malaysia (USM) at a temperature of 26 °C to 32 °C.

A fungal mycelial plug used as an inoculum was prepared from a 7-day-old PDA culture using a sterile cork borer (5 mm diameter). For control, the PDA plugs without fungal mycelia were prepared from the blank PDA using the same methods. Pathogenicity tests for all fungal isolates were performed twice. The tests were carried out on 84 healthy attached young leaves (84 seedlings), 126 stems (126 seedlings), and 150 detached pods of *T. cacao*. The targeted plant parts were surface-sterilized with 70% ethanol prior to inoculation.

To inoculate 13 fungal isolates on leaves of *T. cacao*, a total of 84 healthy leaves (78 for the fungal treatment and six for the control) from 84 seedlings of *T. cacao* were used for two pathogenicity tests. Each surface-sterilized leaf was aseptically pricked at one point with a sterile toothpick represented a replicate. For each pathogenicity test, three replicates were performed for each fungal isolate, using three different leaves from three different seedlings. Controls were performed in the same ways but treated with the blank PDA plugs. A sterile scalpel was used to inoculate control and mycelial plugs onto the control and treatment points, respectively. The plugs were wrapped in sterile cotton wool and fixed to the leaf with cellophane tape to avoid dryness. Each inoculated leaf was covered in a sterile zip lock bag. The inoculated seedlings were kept in the plant house of the School of Biological Sciences, USM for 9 days at temperatures ranging from 26 to 32 °C.

A total of 126 healthy stems of *T. cacao* (126 seedlings) were used to inoculate 20 fungal isolates for twice pathogenicity tests. A small wound (0.5 cm) was created on the sterilized surface of each stem by removing the bark with a sterile scalpel. For each pathogenicity test, three wounded stems from three different seedlings were used to inoculate each fungal isolate, representing triplicates. Control was treated similarly using the blank PDA plugs. Using a sterile scalpel, the mycelial and control plugs were placed on the wounded points, with the mycelium positioned towards the cambium. The moisture of the plugs was maintained by wrapping in sterilized cotton and sealing with parafilm. All the inoculated seedlings were incubated in the plant house of the School of Biological Sciences, USM at temperatures ranging from 26 to 32 °C.

Twice pathogenicity tests conducted on healthy detached cocoa pods involved 150 pods (144 for the fungal treatment and six pods for the control). Control and fungal treatments were inoculated on different pods to avoid symptoms overlapping if both were performed on the same pods. For each pathogenicity test, a wound point was created on the three different pods for each fungal isolate by piercing the pod surface with a sterile cork borer. Then, 5 mm mycelial plugs with the mycelium facing the surface of the pods were placed on the wounded points. The three control pods were treated in the same way but using the blank PDA plugs. To retain moisture, all the plugs were wrapped with sterilized cotton wool and the cotton was fixed with cellophane tape. The inoculated cocoa pods were incubated for 12 days at 25 °C ± 2 °C in sterilized trays and covered with transparent plastic to maintain humidity.

The area of the lesion developed on the inoculated leaves, stems, and pods of *T. cacao* was measured using grid paper adopted by Parker et al.^74^ with slight modifications. The area of diseased lesion was calculated by multiplying the number of small squares covering the lesion with the value calculated for one small square. Differences in lesion area were evaluated using the one-way method ANOVA and means were compared with the Tukey’s test (p < 0.05) using the software IBM SPSS Statistics version 26. To confirm Koch's postulates, fungi from symptomatic inoculated leaves, stems, and pods of *T. cacao* were reisolated and reidentified using morphological characteristics.

## Supplementary Information

Supplementary Figure S1.

## Data Availability

All sequence data are available in NCBI GenBank [https://www.ncbi.nlm.nih.gov/genbank/] following the accession numbers [OL831055–OL831111 (ITS); OL863319–OL863375 (*tef1-α*); OL863262–OL863318 (*tub2*); OL863376–OL863432 (*rpb2*)] in the manuscript. All data analyzed during this study are included in this published article and its supplementary information files.
