# Supplementary material for: Lasiodiplodia theobromae as a causal pathogen of leaf blight, stem canker, and pod rot of Theobroma cacao in Malaysia
Source: Sci Rep. 2022 May 27;12:8966. doi: 10.1038/s41598-022-13057-9 (PMC9142511; doi:10.1038/s41598-022-13057-9)
Supplement: Supplementary file 1 — Supplementary Figure S1. [file 41598_2022_13057_MOESM1_ESM.pdf]

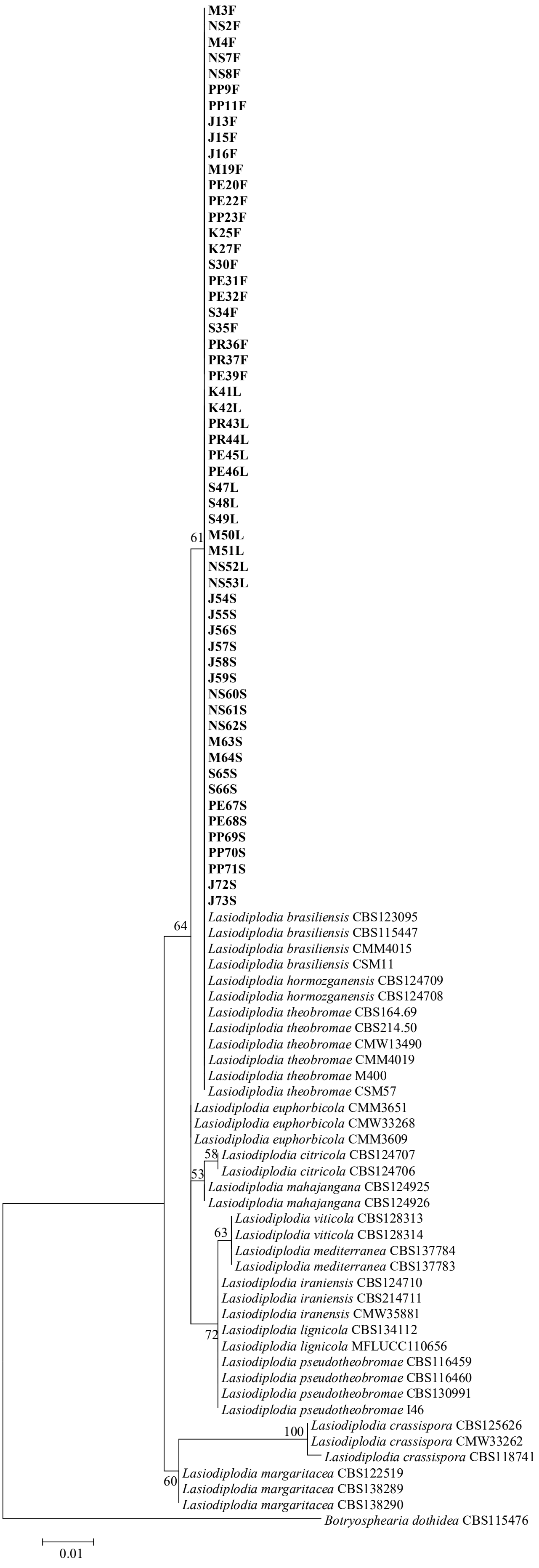

M3F
NS2F
M4F
NS7F
NS8F
PP9F
PP11F
J13F
J15F
J16F
M19F
PE20F
PE22F
PP23F
K25F
K27F
S30F
PE31F
PE32F
S34F
S35F
PR36F
PR37F
PE39F
K41L
K42L
PR43L
PR44L
PE45L
PE46L
S47L
S48L
S49L
61
M50L
M51L
NS52L
NS53L
J54S
J55S
J56S
J57S
J58S
J59S
NS60S
NS61S
NS62S
M63S
M64S
S65S
S66S
PE67S
PE68S
PP69S
PP70S
PP71S
J72S
J73S
Lasiodiplodia brasiliensis CBS123095
64
Lasiodiplodia brasiliensis CBS115447
Lasiodiplodia brasiliensis CMM4015
Lasiodiplodia brasiliensis CSM11
Lasiodiplodia hormozganensis CBS124709
Lasiodiplodia hormozganensis CBS124708
Lasiodiplodia theobromae CBS164.69
Lasiodiplodia theobromae CBS214.50
Lasiodiplodia theobromae CMW13490
Lasiodiplodia theobromae CMM4019
Lasiodiplodia theobromae M400
Lasiodiplodia theobromae CSM57
Lasiodiplodia euphorbicola CMM3651
Lasiodiplodia euphorbicola CMW33268
Lasiodiplodia euphorbicola CMM3609
58
Lasiodiplodia citricola CBS124707
Lasiodiplodia citricola CBS124706
53
Lasiodiplodia mahajangana CBS124925
Lasiodiplodia mahajangana CBS124926
Lasiodiplodia viticola CBS128313
63
Lasiodiplodia viticola CBS128314
Lasiodiplodia mediterranea CBS137784
Lasiodiplodia mediterranea CBS137783
Lasiodiplodia iraniensis CBS124710
Lasiodiplodia iraniensis CBS214711
Lasiodiplodia iranensis CMW35881
72
Lasiodiplodia lignicola CBS134112
Lasiodiplodia lignicola MFLUCC110656
Lasiodiplodia pseudotheobromae CBS116459
Lasiodiplodia pseudotheobromae CBS116460
Lasiodiplodia pseudotheobromae CBS130991
Lasiodiplodia pseudotheobromae I46
Lasiodiplodia crassispora CBS125626
100
Lasiodiplodia crassispora CMW33262
Lasiodiplodia crassispora CBS118741
Lasiodiplodia margaritacea CBS122519
60
Lasiodiplodia margaritacea CBS138289
Lasiodiplodia margaritacea CBS138290
Botryosphearia dothidea CBS115476
0.01

Supplementary Figure S1a. Single gene phylogenetic maximum likelihood (ML) tree of internal transcribed spacer (ITS) sequence using the Kimura-2-parameter model with 1000 bootstrap replications. Bootstrap support values greater than 50% are pointed out at the nodes. Isolates in bold represent isolates in the present study and *Botryosphearia dothidea* represents an outgroup. The bar indicates the substitutions number per position.

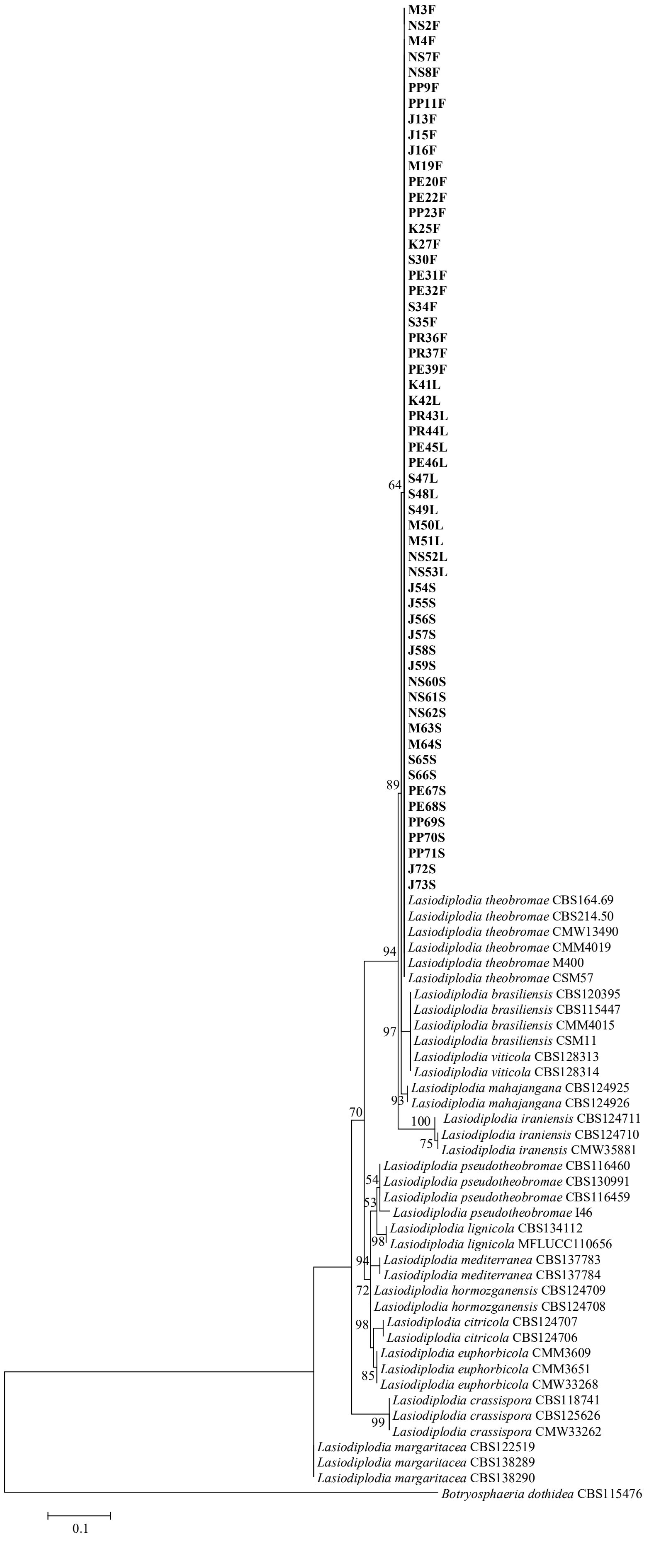

M3F
NS2F
M4F
NS7F
NS8F
PP9F
PP11F
J13F
J15F
J16F
M19F
PE20F
PE22F
PP23F
K25F
K27F
S30F
PE31F
PE32F
S34F
S35F
PR36F
PR37F
PE39F
K41L
K42L
PR43L
PR44L
PE45L
PE46L
S47L
S48L
S49L
M50L
M51L
NS52L
NS53L
J54S
J55S
J56S
J57S
J58S
J59S
NS60S
NS61S
NS62S
M63S
M64S
S65S
S66S
PE67S
PE68S
PP69S
PP70S
PP71S
J72S
J73S
64
89
94
97
93
100
75
70
54
53
98
94
72
98
85
99
Lasiodiplodia theobromae CBS164.69
Lasiodiplodia theobromae CBS214.50
Lasiodiplodia theobromae CMW13490
Lasiodiplodia theobromae CMM4019
Lasiodiplodia theobromae M400
Lasiodiplodia theobromae CSM57
Lasiodiplodia brasiliensis CBS120395
Lasiodiplodia brasiliensis CBS115447
Lasiodiplodia brasiliensis CMM4015
Lasiodiplodia brasiliensis CSM11
Lasiodiplodia viticola CBS128313
Lasiodiplodia viticola CBS128314
Lasiodiplodia mahajangana CBS124925
Lasiodiplodia mahajangana CBS124926
Lasiodiplodia iraniensis CBS124711
Lasiodiplodia iraniensis CBS124710
Lasiodiplodia iranensis CMW35881
Lasiodiplodia pseudotheobromae CBS116460
Lasiodiplodia pseudotheobromae CBS130991
Lasiodiplodia pseudotheobromae CBS116459
Lasiodiplodia pseudotheobromae I46
Lasiodiplodia lignicola CBS134112
Lasiodiplodia lignicola MFLUCC110656
Lasiodiplodia mediterranea CBS137783
Lasiodiplodia mediterranea CBS137784
Lasiodiplodia hormozganensis CBS124709
Lasiodiplodia hormozganensis CBS124708
Lasiodiplodia citricola CBS124707
Lasiodiplodia citricola CBS124706
Lasiodiplodia euphorbicola CMM3609
Lasiodiplodia euphorbicola CMM3651
Lasiodiplodia euphorbicola CMW33268
Lasiodiplodia crassispora CBS118741
Lasiodiplodia crassispora CBS125626
Lasiodiplodia crassispora CMW33262
Lasiodiplodia margaritacea CBS122519
Lasiodiplodia margaritacea CBS138289
Lasiodiplodia margaritacea CBS138290
Botryosphaeria dothidea CBS115476
0.1

Supplementary Figure S1b. Single gene phylogenetic maximum likelihood (ML) tree of translation elongation factor 1-alpha (*tef1-α*) sequence using the Tamura-3-parameter model with 1000 bootstrap replications. Bootstrap support values greater than 50% are pointed out at the nodes. Isolates in bold represent isolates in the present study and *Botryosphearia dothidea* represents an outgroup. The bar indicates the substitutions number per position.

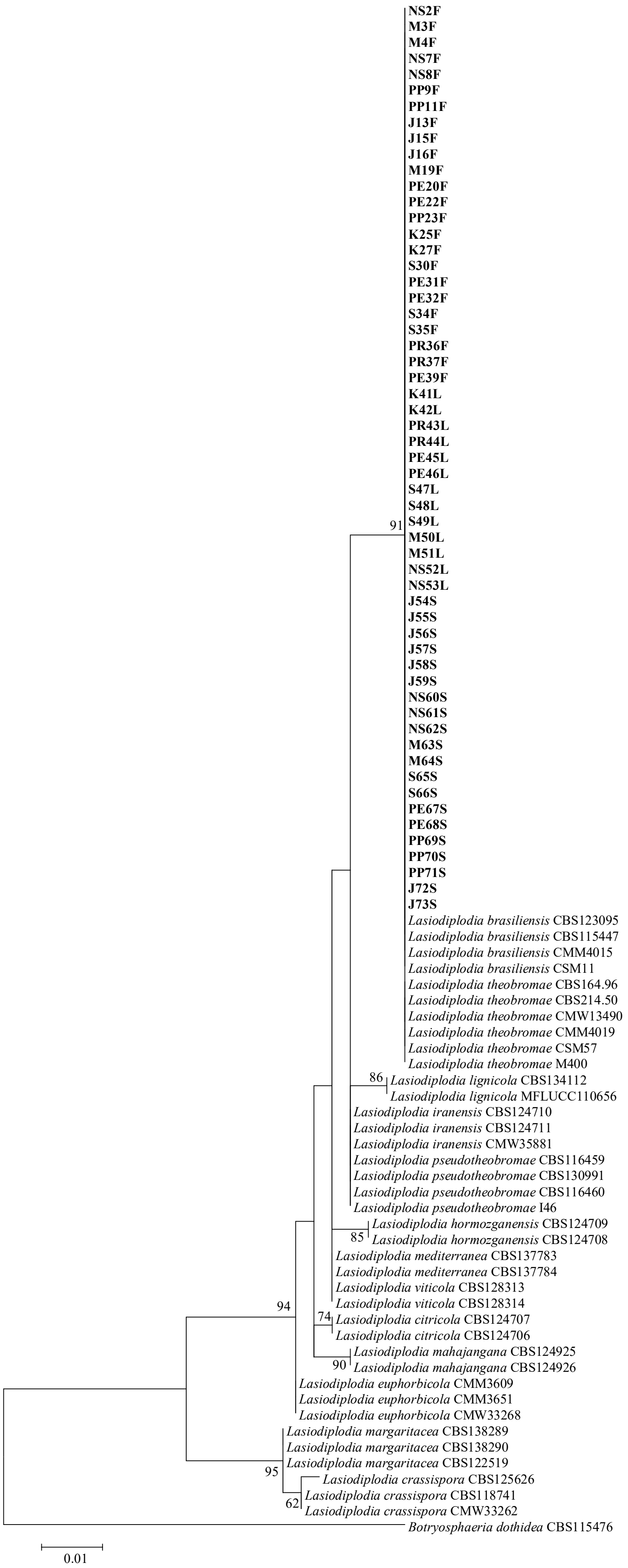

NS2F
M3F
M4F
NS7F
NS8F
PP9F
PP11F
J13F
J15F
J16F
M19F
PE20F
PE22F
PP23F
K25F
K27F
S30F
PE31F
PE32F
S34F
S35F
PR36F
PR37F
PE39F
K41L
K42L
PR43L
PR44L
PE45L
PE46L
S47L
S48L
S49L
91
M50L
M51L
NS52L
NS53L
J54S
J55S
J56S
J57S
J58S
J59S
NS60S
NS61S
NS62S
M63S
M64S
S65S
S66S
PE67S
PE68S
PP69S
PP70S
PP71S
J72S
J73S
Lasiodiplodia brasiliensis CBS123095
Lasiodiplodia brasiliensis CBS115447
Lasiodiplodia brasiliensis CMM4015
Lasiodiplodia brasiliensis CSM11
Lasiodiplodia theobromae CBS164.96
Lasiodiplodia theobromae CBS214.50
Lasiodiplodia theobromae CMW13490
Lasiodiplodia theobromae CMM4019
Lasiodiplodia theobromae CSM57
Lasiodiplodia theobromae M400
86
Lasiodiplodia lignicola CBS134112
Lasiodiplodia lignicola MFLUCC110656
Lasiodiplodia iranensis CBS124710
Lasiodiplodia iranensis CBS124711
Lasiodiplodia iranensis CMW35881
Lasiodiplodia pseudotheobromae CBS116459
Lasiodiplodia pseudotheobromae CBS130991
Lasiodiplodia pseudotheobromae CBS116460
Lasiodiplodia pseudotheobromae I46
Lasiodiplodia hormozganensis CBS124709
85
Lasiodiplodia hormozganensis CBS124708
Lasiodiplodia mediterranea CBS137783
Lasiodiplodia mediterranea CBS137784
Lasiodiplodia viticola CBS128313
Lasiodiplodia viticola CBS128314
94
74
Lasiodiplodia citricola CBS124707
Lasiodiplodia citricola CBS124706
Lasiodiplodia mahajangana CBS124925
90
Lasiodiplodia mahajangana CBS124926
Lasiodiplodia euphorbicola CMM3609
Lasiodiplodia euphorbicola CMM3651
Lasiodiplodia euphorbicola CMW33268
Lasiodiplodia margaritacea CBS138289
Lasiodiplodia margaritacea CBS138290
Lasiodiplodia margaritacea CBS122519
95
Lasiodiplodia crassispora CBS125626
62
Lasiodiplodia crassispora CBS118741
Lasiodiplodia crassispora CMW33262
Botryosphaeria dothidea CBS115476
0.01

Supplementary Figure S1c. Single gene phylogenetic maximum likelihood (ML) tree of β-tubulin (*tub2*) sequence using the Kimura-2-parameter model with 1000 bootstrap replications. Bootstrap support values greater than 50% are pointed out at the nodes. Isolates in bold represent isolates in the present study and *Botryosphearia dothidea* represents an outgroup. The bar indicates the substitutions number per position.

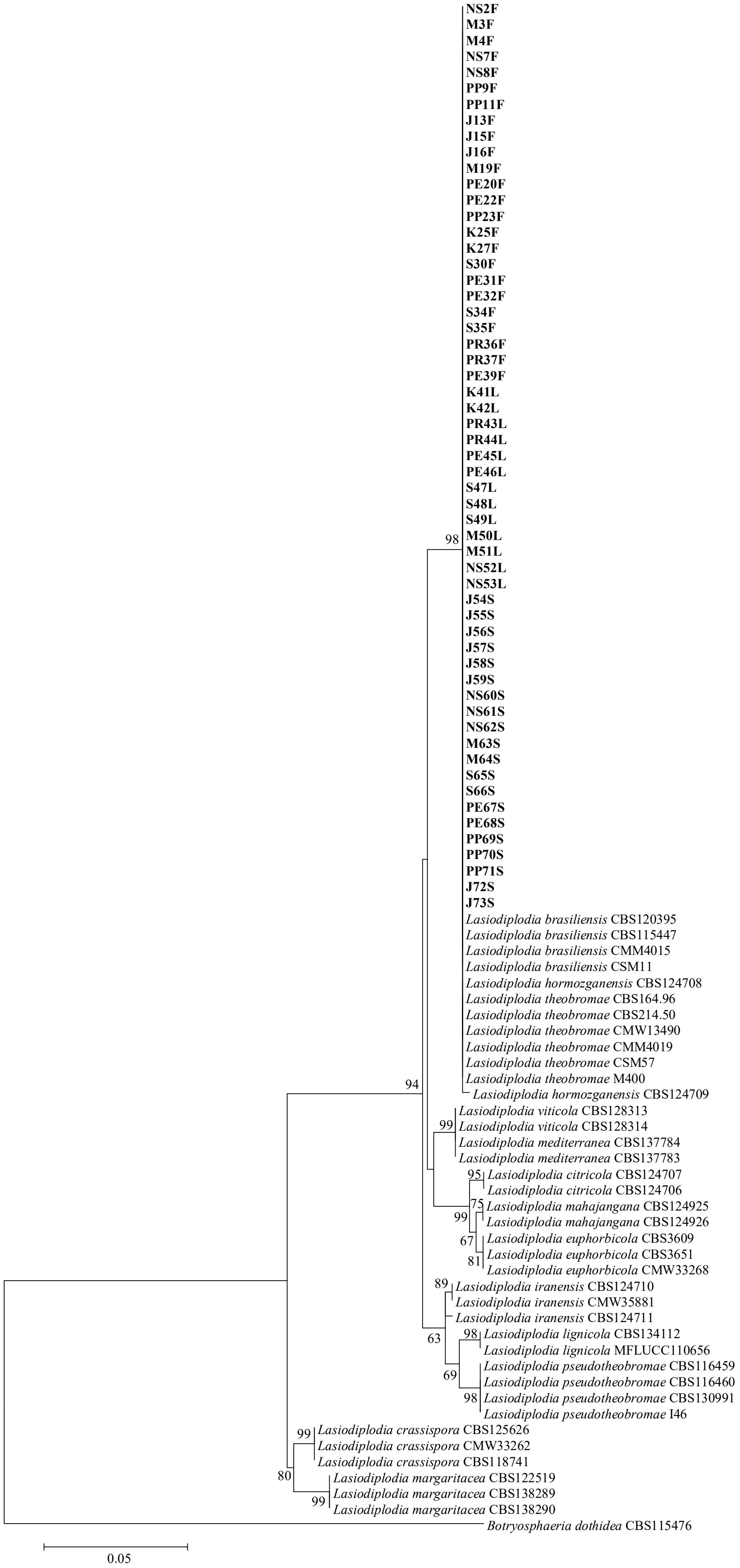
NS2F
M3F
M4F
NS7F
NS8F
PP9F
PP11F
J13F
J15F
J16F
M19F
PE20F
PE22F
PP23F
K25F
K27F
S30F
PE31F
PE32F
S34F
S35F
PR36F
PR37F
PE39F
K41L
K42L
PR43L
PR44L
PE45L
PE46L
S47L
S48L
S49L
98
M50L
M51L
NS52L
NS53L
J54S
J55S
J56S
J57S
J58S
J59S
NS60S
NS61S
NS62S
M63S
M64S
S65S
S66S
PE67S
PE68S
PP69S
PP70S
PP71S
J72S
J73S
Lasiodiplodia brasiliensis CBS120395
Lasiodiplodia brasiliensis CBS115447
Lasiodiplodia brasiliensis CMM4015
Lasiodiplodia brasiliensis CSM11
Lasiodiplodia hormozganensis CBS124708
Lasiodiplodia theobromae CBS164.96
Lasiodiplodia theobromae CBS214.50
Lasiodiplodia theobromae CMW13490
Lasiodiplodia theobromae CMM4019
Lasiodiplodia theobromae CSM57
Lasiodiplodia theobromae M400
94
Lasiodiplodia hormozganensis CBS124709
Lasiodiplodia viticola CBS128313
99
Lasiodiplodia viticola CBS128314
Lasiodiplodia mediterranea CBS137784
Lasiodiplodia mediterranea CBS137783
95
Lasiodiplodia citricola CBS124707
Lasiodiplodia citricola CBS124706
75
Lasiodiplodia mahajangana CBS124925
99
Lasiodiplodia mahajangana CBS124926
67
Lasiodiplodia euphorbicola CBS3609
81
Lasiodiplodia euphorbicola CBS3651
Lasiodiplodia euphorbicola CMW33268
89
Lasiodiplodia iranensis CBS124710
Lasiodiplodia iranensis CMW35881
Lasiodiplodia iranensis CBS124711
98
Lasiodiplodia lignicola CBS134112
63
Lasiodiplodia lignicola MFLUCC110656
Lasiodiplodia pseudotheobromae CBS116459
69
Lasiodiplodia pseudotheobromae CBS116460
98
Lasiodiplodia pseudotheobromae CBS130991
Lasiodiplodia pseudotheobromae I46
99
Lasiodiplodia crassispora CBS125626
Lasiodiplodia crassispora CMW33262
Lasiodiplodia crassispora CBS118741
80
Lasiodiplodia margaritacea CBS122519
Lasiodiplodia margaritacea CBS138289
99
Lasiodiplodia margaritacea CBS138290
Botryosphaeria dothidea CBS115476
0.05

Supplementary Figure S1d. Single gene phylogenetic maximum likelihood (ML) tree of RNA polymerase subunit II (*rpb2*) sequence using the Kimura-2-parameter model with 1000 bootstrap replications. Bootstrap support values greater than 50% are pointed out at the nodes. Isolates in bold represent isolates in the present study and *Botryosphearia dothidea* represents an outgroup. The bar indicates the substitutions number per position.
